# A Retrospective Analysis of the Effectiveness and Safety of Collagen Mesotherapy in the Course of Chronic Cervical Myofascial Pain Syndrome

**DOI:** 10.3390/biomedicines13081893

**Published:** 2025-08-04

**Authors:** Kamil Koszela, Marta Woldańska-Okońska, Barbara Stypińska, Robert Gasik

**Affiliations:** 1 Department of Neuroorthopedics and Neurology Clinic and Polyclinic, National Institute of Geriatrics, Rheumatology and Rehabilitation in Warsaw, 02-637 Warsaw, Poland; 2 Department of Internal Medicine, Rehabilitation and Physical Medicine, Medical University of Lodz, 90-419 Lodz, Poland; 3 Department of Molecular Biology, National Institute of Geriatrics, Rheumatology and Rehabilitation, Spartanska 1, 02-637 Warsaw, Poland

**Keywords:** pain, spine, injection, collagen type I, lignocaine

## Abstract

**Background/Objectives**: Chronic cervical myofascial pain syndrome (CMPS) is often diagnosed in the current population by doctors of various specialties. One method of treating spinal pathology is mesotherapy. The purpose of this study is to evaluate the efficacy and safety of collagen mesotherapy, as well as to assess the frequency of pain medication after mesotherapy in chronic CMPS. **Methods**: Patients were diagnosed and treated by an orthopedist in three different offices between 1 January 2018 and 31 December 2024. The patients were diagnosed with chronic CMPS. Patients were qualified for cervical spine mesotherapy, which was performed weekly, in five repetitions. Retrospectively, based on medical records and in accordance with inclusion and exclusion criteria, two groups were created: group I (*n* = 65) with injectable type I collagen and group II (*n* = 65) with 1% lignocaine. Patients were evaluated using the VAS and Laitinen scale before the start of therapy, 1 week after the end of therapy, and at 3-month follow-up. In addition, the frequency of taking analgesic medications after mesotherapy was assessed. **Results**: After mesotherapy of the cervical spine with both injectable collagen type I and lignocaine 1%, statistically significant improvements were observed in terms of a decrease in pain on the scales used (*p* < 0.001), as well as a decrease in analgesic medication intake (*p* < 0.001). Collagen treatment yielded better results after 3 months of follow-up. No mesotherapy-related side effects were observed during the treatment or follow-up periods. **Conclusions**: Cervical spine mesotherapy using injectable type I collagen and lignocaine 1% is an effective and safe method for chronic CMPS. At a 3-month follow-up, injectable type I collagen appears to be more effective. After mesotherapy and at the 3-month follow-up, both groups reported less pain medication intake compared to before the intervention.

## 1. Introduction

Due to increasing life expectancy, the number of patients with musculoskeletal pathologies, including chronic cervical myofascial pain syndrome (CMPS), among others, is increasing [1]. An unergonomic workplace and excessive and inappropriate use of electronic devices leads to overloading of the cervical spine [2,3]. Excessive strain or injury to the neck muscles, as well as stress and posture-related mechanisms, can lead to myofascial pain in the neck [1]. A common first symptom is increased tension in the soft tissues of the cervical spine, especially the muscles and fascia [4]. Patients have so-called trigger points in tense muscle bands. Latent trigger points are caused by several factors, including stress or changes in posture. New trigger points can also develop after injury or trauma. The currently accepted theory is that there is abnormal growth of acetylcholine in the tissues, leading to increased muscle tension and the formation of tight bands that constrict blood vessels, which activates nociceptors [1]. Fluid retention occurs in tense tissues, as observed using elastography [5]. Because of impaired circulation of this area, the process of healing and self-repair is hindered [6]. As a consequence, there is a restriction of spinal mobility and complaints of pain [7]. Initially, no clinically significant changes are observed in radiological images, except for the loss of cervical lordosis [8]. This results in a lowering of the quality of life, makes it difficult to perform basic activities, and is a cause of absenteeism from work [9].

In the treatment of spinal disorders, there is increasing talk of the so-called three-stage treatment concept, which is multidirectional and consists of the following: 1. assessment of risk factors and their reduction/modification; 2. implementation of medical therapy, conservative or surgical; and 3. implementation of physiotherapy as the third stage [10].

Patients often expect minimally invasive, effective, and safe therapies, which forces medics to look for new therapeutic solutions. One method is mesotherapy, called local intradermal therapy (LIT) [11]. This technique involves multipoint microinjections within the pathologically altered organ. Injections are performed intradermally, at a 15–30-degree angle, using specialized needles 4–13 mm long and 0.23–0.3 mm in diameter (32–30G) [12,13]. Various drugs and medical devices are used, such as ketoprofen [14], dexketoprofen [15], tenoxicam [16], and lignocaine or injectable type I collagen [17,18]. The primary goal of mesotherapy is to trigger the repair mechanisms of the affected area of the musculoskeletal system. An important aspect is the restoration of normal circulation, after which the relaxation of strained tissues occurs, mobility is improved, and pain is reduced [13]. This effect is achieved, among other things, due to irritation of receptors intradermally; the level of endorphins increases and the endogenous opioid system is activated [19,20,21]. Moreover, in addition to the mechanical aspect associated with the injection technique (in this case, mesotherapy), the therapeutic effect is also dependent on the drug or medical device used [13].

The purpose of this study is to evaluate the efficacy and safety of collagen mesotherapy and assess the frequency of taking pain medications after mesotherapy for chronic CMPS.

Hypothesis: Spinal mesotherapy is an effective and safe treatment method and reduces analgesic intake in chronic cervical myofascial pain syndrome.

## 2. Materials and Methods

### 2.1. Study Design

Patients were diagnosed and treated by a single physician (orthopedist) in three different offices between 1 January 2018 and 31 December 2024. After a physical examination, discussion of patients’ history, and diagnostic imaging, some patients were diagnosed with chronic CMPS. Patients who underwent either collagen mesotherapy or lignocaine injection were included in the study. Based on medical records and in accordance with the inclusion and exclusion criteria, two groups of patients were retrospectively created: group I (*n* = 65) with type I collagen for injection and group II (*n* = 65) with 1% lignocaine.

The study was approved by the Bioethics Committee at the National Institute of Geriatrics, Rheumatology and Rehabilitation in Warsaw, Poland (number of approval: KBT-3/10/2023, date of approval: 27 April 2023). In addition, informed consent was obtained from all subjects involved in the study.

### 2.2. Inclusion Criteria

- Chronic cervical myofascial pain syndrome;
- No allergy to injection collagen type I or to lignocaine;
- MRI and/or CT diagnostic images (not older than 6 months).

### 2.3. Exclusion Criteria

- Cervical spinal stenosis;
- Cervical spine injury;
- Scoliosis, spinal anomaly, or other deformity;
- Spinal inflammatory disease or cancer;
- A history of back surgery;
- Using painkillers and/or anti-inflammatory drugs during mesotherapy;
- Receiving regular physiotherapy during mesotherapy and follow-up;
- Lack of patient consent to participate in treatment.

### 2.4. Study Protocol—Rating on Scales

**Figure 1:**
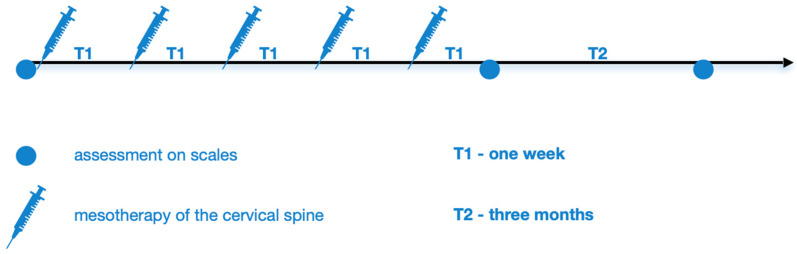
Diagnostic and therapeutic scheme of cervical spinal mesotherapy.

Figure 1 shows the diagnostic and therapeutic scheme for cervical spine mesotherapy. Patients were examined and rated on scales: Visual Analogue Scale (VAS; 0–10) [22] and Latinen scale (0–16), which take into account pain intensity (0–4), pain frequency (0–4), analgesic intake, (0–4), and motor activity limitation (0–4) [23]. This was followed on the same day by a cervical spine mesotherapy treatment, which was performed weekly, in 5 repetitions. Then, 1 week after the fifth mesotherapy treatment and after another 3 months (follow-up period), they were re-evaluated using the above scales at the follow-up visit.

### 2.5. Study Protocol—Mesotherapy Procedure

**Figure 2:**
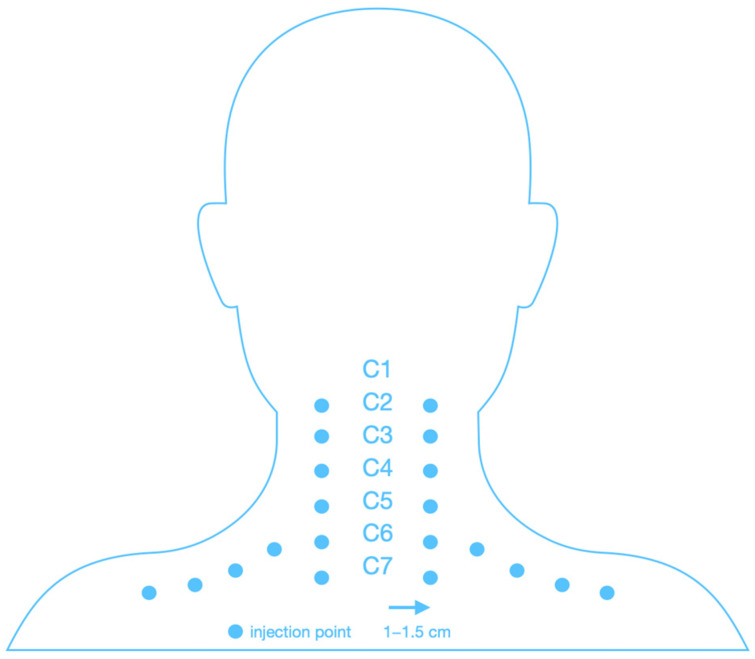
Scheme of injection of the cervical spine.

Figure 2 shows a schematic of the cervical spine mesotherapy procedure. Injections were performed intradermally, at a depth of 3–4 mm and at an angle of 15–30 degrees, using a needle with a diameter of 0.3 mm (30 G) and a length of 12 mm. A total of 20 injections were given during one treatment, and approximately 0.1 mL of the product was administered per injection point (a total of 2 mL of injectable collagen type I or 2 mL of lignocaine 1% during one treatment). Each patient received the same regimen (Figure 2).

### 2.6. Sample Size

Sample size calculation: to achieve 80% power at α = 5% and a normalized effect size of 35%, each group should consist of at least 65 participants [18].

### 2.7. Statistical Analysis

Descriptive statistics were used to summarize patient characteristics at baseline. Continuous variables, such as age, were presented as mean ± standard deviation (SD) and compared between groups using Student’s *t*-test. Ordinal variables, including VAS scores and the Laitinen scale, were expressed as medians and interquartile ranges (1stQ, 3rdQ) and analyzed using the Wilcoxon test. Categorical variables, such as gender distribution and analgesic use, were reported as counts and percentages and assessed using the chi-squared test. A *p*-value < 0.05 was considered statistically significant.

The Cumulative Link Mixed Model (CLMM) was applied to analyze the ordinal dependent variables evaluating the effects of treatment group (collagen type I vs. lignocaine 1%), time points (pre-intervention, post-intervention, 3-month follow-up), and their interaction. A Generalized Mixed Model (GMM) was used to analyze the probability of medication intake over time, taking into account the therapeutic group (type I collagen vs. 1% lignocaine), time points (before intervention, after intervention, 3 months after intervention), and also their interaction.

All statistical analyses and visualizations were performed using R version 4.4.1 (14 June 2024 ucrt) (R Core Team, 2024). The following R packages were utilized for data processing, statistical modeling, and result visualization: readxl, tidyverse, qwraps2, ggstatsplot, ggpubr, rstatix, lme4, lmerTest emmeans, car, ordinal, and knitr [24,25,26,27].

## 3. Results

This retrospective study included 130 patients, consisting of 87 women and 43 men aged from 34 to 68 (Table 1) with chronic CMPS. The diagnosis was confirmed based on a medical examination and evaluation of diagnostic imaging (MRI and/or CT). There was no statistical difference between the groups in terms of age, gender, or initial scale values (Table 1).

**Figure 3:**
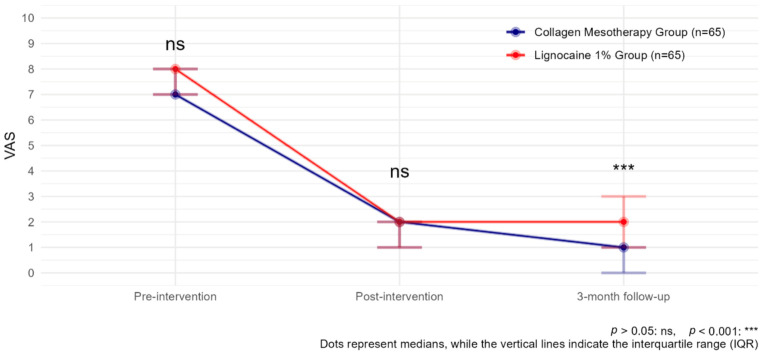
VAS observed with collagen mesotherapy vs. lignocaine 1% at all time points.

Both treatments, collagen mesotherapy and lidocaine mesotherapy, showed significant reductions in pain intensity. The comparison of VAS scores over time showed a significant reduction in pain levels in both groups. Before the intervention, VAS scores were comparable between groups (*p* > 0.05). After the intervention and at the 3-month follow-up, pain levels significantly decreased in both groups compared to baseline (estimate _Post-intervention_ = −10.66, *p* < 0.001; estimate _3-month follow-up_ = −12.73, *p* < 0.001). However, at 3 months, the collagen type I group exhibited a lower VAS score than the lignocaine 1% group (estimate _Lignocaine 1%:time3-month follow-up_ = 2.43, *p* < 0.001), indicating treatment-dependent effects over time (Figure 3, Table 2).

Moreover, both treatments, collagen mesotherapy and lidocaine mesotherapy, showed a significant reduction in pain intensity as measured by the Laitinen scale. The comparison of Laitinen scores over time indicated a statistically significant decrease in pain levels in both groups. Before the intervention, Laitinen scores were comparable between groups (*p* > 0.05). After the intervention, pain levels significantly decreased in both groups compared to baseline (estimate _Post-intervention_ = −9.41, *p* < 0.001). However, at this point, the lignocaine 1% group showed a significantly lower Laitinen score than the collagen type I group (estimate _Lignocaine 1%:timePost-intervention_ = −1.81, *p* = 0.000124), indicating a stronger immediate effect of lidocaine mesotherapy.

**Figure 4:**
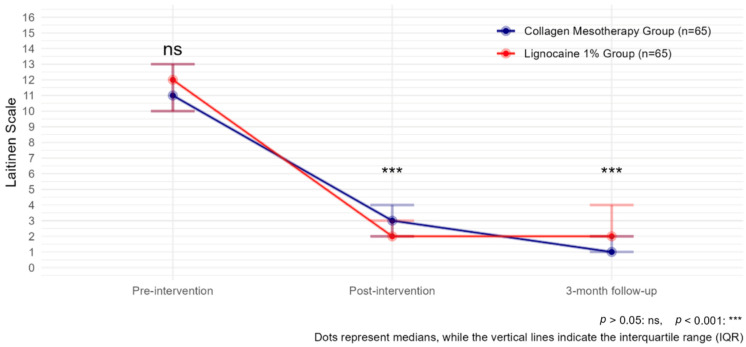
Laitinen scale observed with collagen mesotherapy vs. lignocaine 1% at all time points.

At the 3-month follow-up, pain levels continued to decline (estimate _3-month follow-up_ = −12.39, *p* < 0.001). However, at 3 months, the lignocaine 1% group exhibited a significantly higher Laitinen score than the collagen type I group (estimate _Lignocaine 1%: time 3-month follow-up_ = 2.26, *p* =< 0.001), suggesting that the long-term effect was stronger in the collagen group (Figure 4, Table 3).

Additionally, before starting mesotherapy, 112 patients (86.15%) were taking painkillers. 31 patients (27.7%) used paracetamol, 26 patients (23.2%) used diclofenac, 19 patients (17%) used ketoprofen, 15 patients (13.4%) used dexketoprofen, 10 patients (8.9%) used nimesulide, and 11 patients (9.8%) used tramadol. After the treatment, the number of patients taking medication was reduced to 18 (13.85%), and this number was mostly maintained after 3 months of follow-up, with 13 patients (10%) who remained on pain medication.

**Figure 5:**
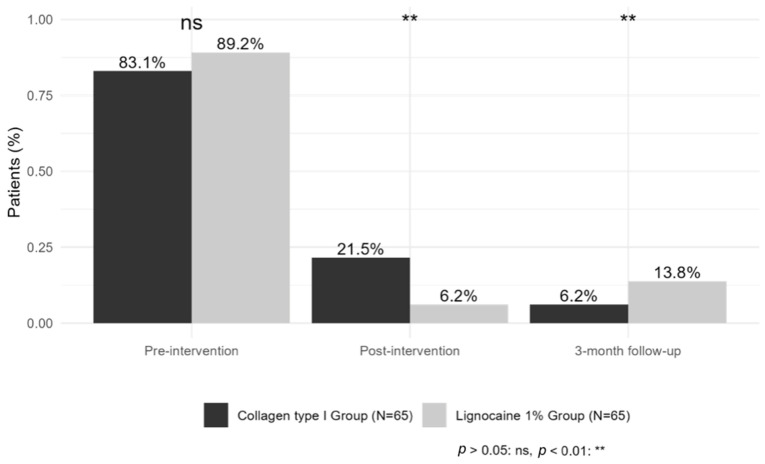
Percentage of patients using analgesic medication.

Pain medication intake probability significantly decreased over time in both groups, with similar baseline values (*p* > 0.05). After the intervention, medication intake was lower compared to pre-intervention in both groups (estimate _Post-intervention_ = −18.40, *p* < 0.001), with the lignocaine 1% group showing a stronger immediate reduction compared to the collagen type I group (estimate _Lignocaine 1%:time post-intervention_ = −7.91, *p* = 0.007). At the 3-month follow-up, medication intake probability remained reduced, though the lignocaine 1% group had a significantly higher probability of intake compared to the collagen type I group (estimate _Lignocaine 1%:time 3-month follow-up_ = 6.55, *p* = 0.007), suggesting a greater long-term impact of collagen therapy (Figure 5).

During the entire therapy, no serious side effects related to mesotherapy were observed. No allergic reactions were observed. The pain associated with mesotherapy disappeared faster after lignocaine than after collagen.

## 4. Discussion

### 4.1. Discussion

This is the first study that presents the efficacy and safety of collagen mesotherapy for chronic cervical myofascial pain syndrome. Based on the published literature to date, the amount of research on collagen mesotherapy of the spine is small and insufficient. Therefore, the present study plays an important role and provides new data.

The present results show a decrease in pain on both the VAS and Laitinen scale at the end of therapy and at 3-month follow-up using injectable collagen type I and lignocaine 1% mesotherapy techniques in chronic CMPS. However, a better therapeutic effect was observed after the use of collagen mesotherapy at the 3-month follow-up. Similar results have been published for chronic low back pain (CLBP) [18] and chronic thoracic back pain [17]. Patients were also evaluated on the VAS and Laitinen scale, and the follow-up period was 3 months, after which better effects were found in favor of collagen mesotherapy. This effect may be related to the activation of tissue regeneration mechanisms after collagen mesotherapy.

After forming, the fibers are stabilized through cross-linking. The final bioactivity of collagen scaffolds is the result of both of these processes. Taking into account each stage of production, scaffolds can be tailored to the specific needs of each tissue, increasing their therapeutic potential [28].

The restoration of the biological scaffold as a result of the external supply of type I collagen, the stimulation of endogenous type I collagen production, and the inhibition of type I collagen degradation by MMP-I may be the reasons for the improved results. Nevertheless, this issue requires further research on a molecular basis [29,30].

In addition, the results presented here show for the first time the aspect of lower use of oral pharmacotherapy after cervical spine mesotherapy. Analgesic and anti-inflammatory drugs are widely used by doctors of various specialties for spinal pain syndrome [31]. Nevertheless, their effectiveness is questionable, especially in chronic pain syndrome [32]. It is worth noting the side effects after pharmacotherapy in a group of geriatric patients, where spinal mesotherapy is an alternative treatment [33].

In recent years, a number of studies have been published demonstrating the effectiveness of mesotherapy for cervical spine pain syndrome. Ranieri et al. [34] in 2024 published an article that also demonstrated the efficacy and safety of mesotherapy with diclofenac in the course of bilateral cervical spine pain syndrome with radiation to the upper extremities. In their study, in addition to pain assessment (on the Visual Analogue Scale VAS), range of movement (ROM) and the bilateral trapezius’ dynamic stiffness were measured using MyotonPro, Myoton AS, Tallinn, Estonia.

In addition, Scaturro et al. [35] in 2023 published an article demonstrating the efficacy and safety of cervical spine mesotherapy for fibromyalgia. In their study, they used mesotherapy with diclofenac, thiococolchicoside, and mepivacaine compared to a placebo group with sodium chloride. This is the first study showing the use of mesotherapy in combination with movement therapy, which plays an important role in the healing process. The study used Numeric Rating Scales (NRSs), the Fibromyalgia Impact Questionnaire (FIQ), the Neck Disability Index (NDI), and the 12-item Short-Form Survey (SF-12) to assess outcomes. In addition, it is worth mentioning that the treatment of fibromyalgia is difficult, so mesotherapy is an alternative in this group of patients.

It is worth mentioning another study published by Viscito R. et al. [36], where mesotherapy was used in patients affected by neck pain in spondylarthrosis. One group was treated with saline solution and the other with a cocktail of drugs (normal saline solution, lidocaine hydrochloride, and lysine acetylsalicylate). A therapeutic effect was achieved in both groups. However, only patients treated with the cocktail of drugs showed improvement at three months following treatment. The effects were assessed using the Visual Analogue Scale (VAS), the short-form McGill pain questionnaire, the Present Pain Intensity scale, and the Neck Disability Index (NDI).

Based on an analysis of the existing literature, the mesotherapy technique is among the safest methods of minimally invasive therapy (MIT) in the course of musculoskeletal pathologies, both with the use of NSAIDs [37,38] and also injectable type I collagen [17,18]. Nevertheless, the number of publications in this area is still insufficient, which necessitates further research on this topic. Especially desirable are prospective studies designed in accordance with evidence-based medicine (EBM), conducted with an analysis of biochemical parameters of inflammation and pain.

Mesotherapy is an injection technique used by many doctors around the world in the treatment of various musculoskeletal disorders, including the spine. As described above, mesotherapy is a safe procedure. However, as in the case of any medical procedure, there is a risk of complications. The most common are local pain and swelling. Microhematomas sometimes occur. Bacterial or viral infections may develop, causing inflammation and the formation of microabscesses. Therefore, it is essential to prepare properly for the procedure, including disinfecting the treatment area and the doctor’s hands, using sterile gloves, and ensuring that the instruments used (needle, syringe) are also sterile [39].

It is worth noting the latest publication on the international consensus on safety and evidence-based practice in mesotherapy. The era of mesotherapy, which until now has been based on personal empirical experience, is now opening up to a new, science-based approach to practice [40].

### 4.2. Limitations

The fact that the study is retrospective is its main limitation. Nevertheless, the diagnosis and qualification for mesotherapy did not raise any doubts. In addition, despite the restrictive inclusion and exclusion criteria, during the follow-up period, patients could take medications or exercise ad hoc, which could have affected the final results of the study. Patients who declared regular use of painkillers and/or anti-inflammatory drugs or physiotherapy during mesotherapy and follow-up were excluded from the study. In addition, studies with a saline control group are also needed. Another comparison group could be a group in which medical interventions were not performed for any reason. It would also be worthwhile to conduct studies assessing diagnostic imaging (MRI, CT, ultrasound, and elastography) before and after mesotherapy. Therefore, further research is needed, especially prospective, randomized studies with longer follow-up periods.

## 5. Conclusions

Cervical spine mesotherapy using type I collagen for injection and 1% lignocaine is an effective and safe method of treating chronic CMPS. However, after 3 months of observation, type I collagen for injection appears to be more effective. Furthermore, right after mesotherapy and after 3 months of observation, both groups reported lower consumption of analgesics compared to the period before the procedure.

## Figures and Tables

**Table 1:** Patient characteristics at baseline.

	Collagen Type I Group (N = 65)	Lignocaine 1% Group (N = 65)	*p*-Value
Female, *n* (%)	46 (70.77%)	41 (63.08%)	0.46 ^1^
Male, *n* (%)	19 (29.23%)	24 (36.92%)	
Age, mean ± SD	49.80 ± 8.84	50.17 ± 8.95	0.81 ^2^
Pre-therapy VAS, median (1stQ, 3rdQ)	7.00 (7.00, 8.00)	8.00 (7.00, 8.00)	0.26 ^3^
Pre-therapy Laitinen scale, median (1stQ, 3rdQ)	11.00 (10.00, 13.00)	12.00 (10.00, 13.00)	0.56 ^3^
Pre-therapy analgesic use, *n* (%)	54 (83.08%)	58 (89.23%)	0.45 ^1^

^1^ chi-squared test; ^2^ Student’s *t*-test; ^3^ Wilcoxon test; 1stQ, 3rdQ: first quartile third quartile; SD: standard deviation.

**Table 2:** VAS: mean (SE) by group and time point.

Group	VAS Before	VAS After	VAS 3 m	Δ Post–Baseline	Δ Follow-Up–Baseline	Δ Follow-Up–Post
Collagen type I	7.38 (0.14)	1.80 (0.16)	0.91 (0.09)	−5.58 (0.17)	−6.48 (0.15)	−0.89 (0.12)
Lignocaine 1%	7.60 (0.14)	1.60 (0.10)	2.08 (0.12)	−6.00 (0.16)	−5.52 (0.18)	0.48 (0.14)
			Diffrence (SE)	−0.42 (0.24)	0.95 (0.19)	1.37 (0.19)
			95% CI	−0.88, 0.05	0.49, 1.42	1.00, 1.74
			*p*-value	ns	<0.001	<0.001

Mean (SE) Visual Analog Scale (VAS) pain intensity scores by treatment group and time point, including mean changes and between-group comparisons. VAS = Visual Analog Scale; SE = Standard Error; Δ = change from baseline. The table shows mean pain scores at baseline (before intervention), immediately post-intervention, and at 3-month follow-up for the collagen type I and lignocaine 1% groups. Mean changes (Δ) represent differences between time points (post–baseline, follow-up–baseline, follow-up–post). Between-group differences with 95% confidence intervals (CI) and corresponding *p*-values are included. *p*-values were obtained from linear mixed-effects models comparing group differences in change scores.

**Table 3:** Laitinen: mean (SE) by group and time point.

Group	Laitinen Before	Laitinen After	Laitinen 3 m	Δ Post–Baseline	Δ Follow-Up–Baseline	Δ Follow-Up–Post
Collagen type I	11.23 (0.25)	3.26 (0.22)	1.71 (0.14)	−7.97 (0.24)	−9.52 (0.24)	−1.55 (0.15)
Lignocaine 1%	11.43 (0.27)	2.25 (0.13)	2.85 (0.16)	−9.18 (0.27)	−8.58 (0.31)	0.60 (0.16)
			Diffrence (SE)	−1.22 (0.37)	0.94 (0.39)	2.15 (0.22)
			95% CI	−1.94, −0.49	0.16, 1.71	1.72, 2.59
			*p*-value	0.001	0.018	<0.001

Mean (SE) Laitinen pain scores by treatment group and time point, with mean changes and group comparisons. Laitinen score = pain intensity measure; SE = Standard Error; Δ = change from baseline. This table presents mean pain scores at baseline, post-intervention, and 3-month follow-up in the collagen type I and lignocaine 1% groups. Changes (Δ) represent within-group differences between time points. Between-group differences with 95% confidence intervals (CI) and *p*-values are shown. *p*-values were calculated using linear mixed-effects models assessing differences in change scores between treatment groups.

## Data Availability

The data are available from the corresponding author if required.
